# ABO Blood Group and Incidence of Skin Cancer

**DOI:** 10.1371/journal.pone.0011972

**Published:** 2010-08-04

**Authors:** Jing Xie, Abrar A. Qureshi, Yunhui Li, Jiali Han

**Affiliations:** 1 Department of Epidemiology, Harvard School of Public Health, Boston, Massachusetts, United States of America; 2 Clinical Research Program, Department of Dermatology, Brigham and Women's Hospital, Harvard Medical School, Boston, Massachusetts, United States of America; 3 Channing Laboratory, Department of Medicine, Brigham and Women's Hospital and Harvard Medical School, Boston, Massachusetts, United States of America; Women's College Research Institute, University of Toronto, Canada

## Abstract

**Background:**

Previous studies have examined the association between ABO blood group and the risk of some malignancies. However, no prospective cohort study to date has examined the association between ABO blood group and the risk of skin cancer.

**Methodology/Principal Findings:**

Using two large cohorts in the US, we examined ABO blood type and incidence of skin cancer, including melanoma, squamous cell carcinoma (SCC), and basal cell carcinoma (BCC). We followed up study participants (70,650 female nurses and 24,820 male health professionals) on their diagnosis of incident skin cancer from cohort baseline (1976 in women and 1986 in men) until 2006. Study participants reported their blood type in 1996 in both cohorts. During the follow-up, 685 participants developed melanoma, 1,533 developed SCC and 19,860 developed BCC. We used Cox proportional hazards models to calculate the hazard ratios (HR) and 95% confidence intervals (CI) of each type of skin cancer. We observed that non-O blood group (A, AB, and B combined) was significantly associated with a decreased risk of non-melanoma skin cancer overall. Compared to participants with blood group O, participants with non-O blood group had a 14% decreased risk of developing SCC (multivariable HR: 0.86; 95% CI: 0.78, 0.95) and a 4% decreased risk of developing BCC (multivariable HR: 0.96; 95% CI: 0.93, 0.99). The decreased risk of melanoma for non-O blood group was not statistically significant (multivariable HR: 0.91; 95% CI: 0.78, 1.05).

**Conclusion/Significance:**

In two large independent populations, non-O blood group was associated with a decreased risk of skin cancer. The association was statistically significant for non-melanoma skin cancer. Additional studies are needed to confirm these associations and to define the mechanisms by which ABO blood type or closely linked genetic variants may influence skin cancer risk.

## Introduction

Skin cancer is the most common malignancy and represents approximately half of all cancers in the United States. Each year more than 1 million cases of skin cancer are diagnosed in this country. There were about 11,590 deaths from skin cancer in 2009 [1]. Risk factors of skin cancer include ultraviolet light exposure, age, male gender, genetic susceptibility, and constitutional factors, for instance hair color, number of moles, skin color, and skin reaction to sun exposures [1]–[5]. There are three types of skin cancer: melanoma, squamous cell carcinoma (SCC), and basal cell carcinoma (BCC) originating from three major types of cells in the epidermis.

ABO blood groups are defined by carbohydrate moieties on the extracellular surface of the red blood cell membranes. Red blood cell antigens have various functions, including membrane structural integrity, transportation of molecules through membranes, and adhesion [6]. Along with their expression on red blood cells, ABO antigens are also highly expressed on the surface of epithelial cells. Previous studies suggest a possible association between ABO blood group and the risk of some epithelial malignancies, including pancreatic cancer [7] and gastric cancer [8]. Several plausible mechanisms, including inflammation, immune-surveillance for malignant cells, intercellular adhesion, and membrane signaling have been proposed to explain the observed association between ABO blood groups and cancer risk [7].

The purpose of this study was to examine possible associations between ABO blood group and the risk of various types of skin cancer in two large national cohorts.

## Materials and Methods

### Ethics Statement

This study was approved by the Human Research Committee at the Brigham and Women's Hospital (Boston, MA) with written informed consent from all participants.

### Study population

The Nurses' Health Study (NHS) began in 1976, when 121,700 registered nurses aged 30–50 years in 11 US states completed a baseline questionnaire regarding risk factors for cancer and cardiovascular diseases. Participants completed self-administered, mailed follow-up questionnaires biennially with updated information on their lifestyle, medical history, and diet. The Health Professionals Follow-up Study (HPFS) began in 1986 when 51,529 US male health professionals, including dentists, veterinarians, pharmacists, and optometrists, aged 40–75 years, completed a baseline questionnaire on lifestyle, diet, and medical conditions. The information was updated biennially with follow-up questionnaires.

### Assessment of ABO blood group and skin cancer risk factors

In 1996, questions on ABO blood group were included in the questionnaires. Participants in both the NHS and HPFS cohorts were asked about their blood type (A, B, AB, O, or unknown). Though not all ABO blood type results were confirmed serologically, we did obtain laboratory confirmation of the self-reported blood type in a subsample of 98 participants for validation. The overall concordance rate was 91% between the self-reported results and the serologically confirmed results. This rate of validation of ABO blood group did not differ between the two cohorts: the concordance rate was 93% for NHS and 90% for HPFS [7]. For skin cancer risk factors, data were obtained from follow-up questionnaires in both cohorts. They included sunburn reaction, family history of melanoma, number of severe sunburns, number of moles, hair color, sun exposure, and residence at different ages.

### Identification of skin cancer cases

Skin cancer identification was performed routinely in both cohorts. Participants reported new diagnosis biennially. With their permission, participants' medical records were obtained and reviewed by physicians to confirm their self-reported diagnosis. Only pathologically confirmed invasive cases of melanoma and SCC were included in this study. Medical records were not obtained for self-reported cases of BCC, and the validity of BCC self-reports was ≥90% in our study [9], [10].

### Statistical analyses

Participants who did not report their blood type in the 1996 questionnaire were excluded from analyses (44,287 in NHS and 21,360 in HPFS). Non-whites were excluded due to insufficient sample sizes in each race category for analyses. Also excluded were participants who had cancer, including skin cancer, before baseline in both cohorts (1976 in the NHS and 1986 in the HPFS). The primary exposure was the participants' self-reported ABO blood type, assessed in 1996. Participants contributed person-time from the baseline. Accumulation of follow-up time ceased at the first report of BCC, the first report followed by confirmation of SCC, the first report followed by confirmation of melanoma, death from another cause, or the end of follow-up (NHS by June 2006; HPFS by January 2006), whichever came earlier. We used Cox proportional hazards models to calculate the hazard ratios (HRs) and 95% confidence intervals (CIs) of each type of skin cancer. We tested the assumption of Proportional Hazards. The tests were not statistically significant (p = 0.34 for melanoma, p = 0.25 for SCC, and p = 0.79 for BCC). In the multivariate analysis, we simultaneously controlled for the above-mentioned skin cancer risk factors. In the combined analysis of the two cohorts, we additionally controlled for gender.

## Results

There were 70,650 female nurses and 24,820 male health professionals included in the analysis. The mean follow-up time was 27.1 years for women and 16.9 years for men. The characteristics of participants in our study were similar across ABO blood groups (**Table 1**). The frequency distribution of ABO blood groups in the NHS and HPFS were similar (**Table 2**).

**Table 1:** Characteristics of the Nurses' Health Study and Health Professional Follow-Up Study combined by ABO blood type (1996).

	Blood group			
Characteristics	O	A	AB	B
No. of subjects				
NHS	30,298	25,455	5,529	9,368
HPFS	10,609	9,397	1,843	2,971
Total	40,907	34,852	7,372	12,339
Mean age, years	62.3	62.2	63.5	62.1
Female, %	74.1	73.0	75.5	75.7
Red or Blond hair, %	15.5	15.4	14.8	14.7
Adolescent sunburn reaction, %	46.8	45.4	44.3	44.6
Number of severe sunburns 6+, %	48.3	47.8	46.2	47.1
Number of moles on arms 3+, %	13.1	13.0	12.6	12.5
Family history of melanoma, %	2.9	3.0	3.4	3.1
College/high school sun exposure≥11 hrs/week, %	23.2	23.6	21.9	22.0
Age 25–35≥11 hrs/week, %	18.2	18.0	17.4	16.6
Age 36–59≥11 hrs/week, %	14.5	14.0	14.5	13.2
Age 60≥11 hrs/week, %	12.6	12.8	12.8	11.2

**Table 2:** Distribution of ABO blood group and Rh type among subjects from NHS, HPFS, and two cohorts combined.

Blood group	NHS (%)	HPFS (%)	Total study population (%)
O	42.9	42.7	42.8
A	36.0	37.9	36.5
AB	7.8	7.4	7.7
B	13.3	12.0	12.9
Rh negative	22.6	21.4	22.3
Rh positive	77.4	78.6	77.7

NHS = Nurses' Health Study, HPFS = Health Professionals Follow-up Study.

**Figure 1:**
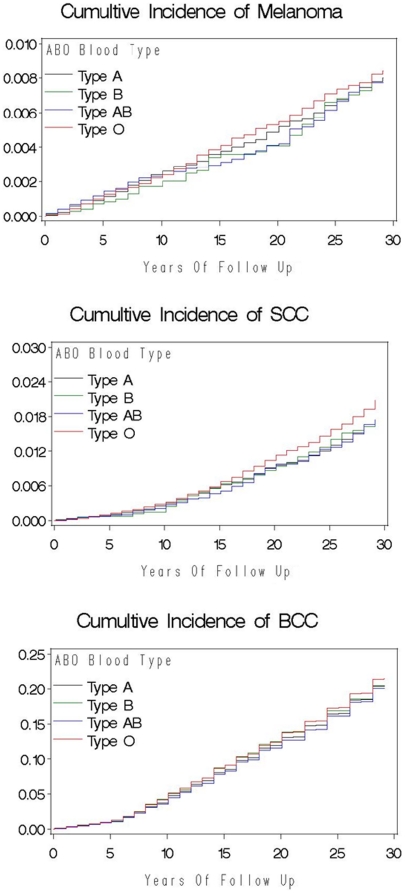
Cumulative Incidence of Melanoma, SCC and BCC.

Between baseline and 2006, 685 participants developed melanoma, 1,533 participants developed SCC, and 19,860 participants developed BCC. Compared to participants with blood group O, the risk of developing SCC was significantly lower for participants in blood group A (multivariable HR: 0.86; 95% CI: 0.77, 0.96). We did not observe a significantly lower risk of developing SCC for participants with blood group AB and B (multivariable HR: 0.83; 95% CI: 0.67, 1.01 and multivariable HR: 0.88; 95% CI: 0.75, 1.04) compared to participants with blood group O. Overall, the risk of developing SCC was significantly lower for participants with non-O blood group (A, AB, and B combined) (multivariable HR: 0.86; 95% CI: 0.78, 0.95) compared to participants with blood group O. The slightly decreased risk of developing BCC was similar across blood groups of A, AB, and B, compared to participants with blood group O. The multivariable HR of BCC was 0.96 (95% CI: 0.93, 0.99) for non-O blood group (A, AB, and B combined) compared to participants with blood group O. We did not observe a statistically significant decreased risk of developing melanoma across blood groups of A, AB, and B, compared to participants with blood group O. The multivariable HR of melanoma for non-O blood group (A, AB, and B combined) was 0.91 (95% CI: 0.78, 1.05). The HRs of each type of skin cancer did not change materially after multivariable adjustment for other risk factors of skin cancer. Kaplan-Meier Curves of melanoma, SCC, and BCC were shown in **Figure 1**. There was no association between the Rh factor and the risk of any type of skin cancer (**Table 3**).

**Table 3:** Age-adjusted^1^ and multivariable-adjusted^2^ hazard ratios and 95% confidence intervals for skin cancers by ABO blood type.

	ABO blood type				
Chracteristics	O	A	AB	B	A, AB, B combined
**Melanoma**					
NHS^1^	1.00 (ref.)	0.86 (0.70–1.05)	0.84 (0.59–1.19)	0.80 (0.59–1.07)	0.84 (0.70–1.00)
NHS^2^	1.00 (ref.)	0.88 (0.72–1.07)	0.87 (0.61–1.24)	0.80 (0.60–1.08)	0.86 (0.72–1.03)
HPFS^1^	1.00 (ref.)	1.02 (0.75–1.39)	0.85 (0.47–1.53)	1.07 (0.69–1.66)	1.01 (0.77–1.34)
HPFS^2^	1.00 (ref.)	1.04 (0.76–1.42)	0.94 (0.52–1.69)	1.12 (0.72–1.75)	1.04 (0.79–1.38)
Combined^1^^,^ 3	1.00 (ref.)	0.91 (0.77–1.07)	0.83 (0.61–1.12)	0.87 (0.68–1.11)	0.89 (0.76–1.03)
Combined^2^^,^ 3	1.00 (ref.)	0.92 (0.78–1.09)	0.88 (0.65–1.19)	0.89 (0.70–1.13)	0.91 (0.78–1.05)
Combined^3^ No. of cases^4^	313	242	48	82	372
**Squamous Cell Carcinoma**					
NHS^1^	1.00 (ref.)	0.85 (0.74–0.99)	0.80 (0.62–1.04)	0.84 (0.68–1.03)	0.84 (0.74–0.96)
NHS^2^	1.00 (ref.)	0.87 (0.75–1.01)	0.86 (0.66–1.11)	0.86 (0.70–1.06)	0.87 (0.76–0.99)
HPFS^1^	1.00 (ref.)	0.84 (0.70–1.00)	0.70 (0.50–0.99)	0.91 (0.70–1.18)	0.84 (0.71–0.98)
HPFS^2^	1.00 (ref.)	0.85 (0.71–1.02)	0.78 (0.55–1.09)	0.93 (0.72–1.21)	0.86 (0.74–1.01)
Combined^1^^,^ 3	1.00 (ref.)	0.86 (0.77–0.96)	0.75 (0.61–0.92)	0.86 (0.73–1.01)	0.84 (0.76–0.93)
Combined^2^^,^ 3	1.00 (ref.)	0.86 (0.77–0.96)	0.83 (0.67–1.01)	0.88 (0.75–1.04)	0.86 (0.78–0.95)
Combined^3^ No. of cases^4^	720	525	104	184	813
**Basal Cell Carcinoma**					
NHS^1^	1.00 (ref.)	0.94 (0.90–0.97)	0.90 (0.84–0.96)	0.92 (0.87–0.97)	0.93 (0.90–0.96)
NHS^2^	1.00 (ref.)	0.95 (0.92–0.99)	0.94 (0.88–1.00)	0.92 (0.87–0.96)	0.94 (0.91–0.97)
HPFS^1^	1.00 (ref.)	0.97 (0.92–1.03)	0.99 (0.89–1.10)	1.00 (0.91–1.09)	0.98 (0.93–1.03)
HPFS^2^	1.00 (ref.)	0.99 (0.93–1.05)	1.09 (0.98–1.21)	1.04 (0.95–1.13)	1.01 (0.96–1.07)
Combined^1^^,^ 3	1.00 (ref.)	0.95 (0.92–0.98)	0.91 (0.87–0.97)	0.94 (0.89–0.98)	0.94 (0.92–0.97)
Combined^2^^,^ 3	1.00 (ref.)	0.96 (0.93–0.99)	0.97 (0.92–1.03)	0.95 (0.90–0.99)	0.96 (0.93–0.99)
Combined^3^ No. of cases^4^	8,758	7,106	1,526	2,470	11,102

1 Age-adjusted.

2 Multivariate-adjusted for sunburn reaction, family history of melanoma, number of severe sunburns, number of moles, hair color, sun exposure, and residence at different ages.

3 The combined analysis of the two cohorts additionally adjusted for gender.

4 Number of person-years: 995,274, 850,292, 180,025, and 305,206 for O, A, AB, and B blood types, respectively.

In addition, we performed a secondary analysis using the ABO blood group assessment date (1996) as the baseline of the follow-up. With 571 SCC cases in total, we confirmed this result: the HR of SCC for participants with blood group A was 0.83 (95% CI: 0.71, 0.95) in the two cohorts combined. For participants with blood group AB and B, the HRs of SCC were 0.85 (95% CI: 0.65, 1.10) and 0.92 (95% CI: 0.75, 1.13) compared to participants with blood group O. No statistically significant associations were observed between any blood group and the risks of melanoma and BCC.

## Discussion

Our results suggest that non-O blood group was significantly associated with a decreased risk of non-melanoma skin cancer overall. Compared to participants with blood group O, participants with non-O blood group had a 14% decreased risk of developing SCC and a 4% decreased risk of developing BCC. The decreased risk of melanoma for non-O blood group (A, AB, and B combined or separately) was not statistically significant.

There are several plausible hypotheses for the observed association between ABO blood groups and skin cancer risk. One possible explanation is that ABO blood group may be directly associated with skin cancer through certain biological mechanisms. Blood group antigens are expressed on the surface of many epithelial cells, including skin cells [11]. One study evaluating normal penile skin compared to squamous cell carcinoma showed less A antigen expression in SCC compared to normal skin [12], which is consistent with our finding that A blood type was less common in SCC cases. Other studies have shown that ABO blood group antigen expression in tumors is associated with metastasis and prognosis [8], [13], [14], [15]. Hence, several plausible mechanisms, including inflammation, immunosurveillance for malignant cells, intercellular adhesion, and membrane signaling have been proposed to explain the observed association between ABO blood groups and cancer risk [7]. In addition, the *ABO* gene on chromosome 9q34 encodes glycotransferases to form the ABO blood group antigens [16]. Differential expression of blood group antigens on epithelial cells may influence tumorigenesis through altered glycosyltransferase specificity [7]. An alternative explanation is that ABO blood group may be indirectly associated with skin cancer. It is possible that the ABO gene was in linkage disequilibrium with other genes involved in skin carcinogenesis.

Many previous studies have reported an association between ABO blood group and the risk of various cancers, especially epithelial cancers. One study using the same two cohorts reported an association between ABO blood type and the risk of developing pancreatic cancer (*P* = .004; log-rank test). In this study, compared with participants with blood group O, those with blood groups A, AB, or B were more likely to develop pancreatic cancer (adjusted HRs for incident pancreatic cancer were1.32, 1.51, and 1.72, respectively) [7]. Another NHS-based study suggested the presence of the B antigen was positively associated with the risk of epithelial ovarian cancer, while blood group A was not associated with risk (manuscript in submission).

To date, ours is the first prospective cohort study investigating the relationship between ABO blood group and the risk of skin cancers. Only one previous case-control study conducted in Turkey examined the relationship between ABO blood groups and skin cancers [17]. The study recruited 98 histologically confirmed skin cancer cases (23 SCC, 42 BCC, and 33 *in situ* SCC) and 419 healthy controls. Skin cancer cases were more likely than controls to be in blood groups A, AB, and B than O (odds ratios ranged from 1.50 to 3.77), but these results were not statistically significant. There are several possible reasons for the observed differences between our results and the results of the previous case-control study. First, our participants are white residing in the US, while the case-control study population was from Mersin in Turkey. The association between ABO blood group and the risk of skin cancer may vary among different races or ethnicities. Second, results from the previous case-control study were based on a relatively small sample size, and confidence intervals were wide.

One of the major strengths of our study is its prospective design. We performed a secondary analysis using the ABO blood group assessment data (1996) as the baseline for the follow-up to confirm our primary analysis results. Another strength of our study is the large study population and the high follow-up rate. Also, the availability of many risk factor covariates allowed us to assess the association while controlling for potential confounding within each ABO blood group. The high concordance rate between reported blood type and serologic testing in a subset of our study population suggests the validity of self-reported blood type.

In conclusion, data from two large cohort studies demonstrated the association between non-O blood group and a decreased risk of each type of skin cancer. The association was statistically significant for non-melanoma skin cancer. The opposing association of the non-O blood group with skin cancer compared to that with pancreatic, gastric, and ovarian cancers suggests unique carcinogenic mechanisms of skin cancer. We considered the analysis exploratory and further investigation is warranted to confirm this finding and to illuminate skin carcinogenesis.
